# Association of thyroid peroxidase antibodies with thyroid nodules and differentiated thyroid cancer in Graves' disease: a retrospective cohort study

**DOI:** 10.20945/2359-4292-2026-0123

**Published:** 2026-10-11

**Authors:** Elif Güneş, Mutlu Güneş, Muhammet İlker Kaya, Murat Yüksel, Sümeyye Meinet, Beyza Kibar, Nizameddin Koca, Gökhan Garip, Behiye Oral

**Affiliations:** 1 Department of Endocrinology, Metabolism and Diabetes, Health Sciences University, Bursa State Hospital, Bursa, Turkey; 2 Department of Endocrinology, Metabolism and Diabetes, Health Sciences University, Bursa Yuksek Ihtisas Training and Research Hospital, Bursa, Turkey; 3 Department of Internal Medicine, Health Sciences University, Bursa State Hospital, Bursa, Turkey; 4 Department of General Surgery, Bursa State Hospital, Bursa, Turkey; 5 Department of Radiology, Bursa State Hospital, Bursa, Turkey

**Keywords:** Graves' disease, thyroid peroxidase antibodies, thyroid nodules, differentiated thyroid cancer, autoimmune thyroid disease, hyperthyroidism

## Abstract

**Objective:**

In Graves' disease (GD), thyroid nodules are the principal determinant of differentiated thyroidcaneer(DTC); however, the predictors of nodules and DTC remain uncertain. The role of thyroid peroxidase antibodies (TPOAb) in this context is controversial. Hence, this study aimed to determine whether TPOAb status is associated with thyroid nodules and DTC in GD and to compare the prevalence of DTC across subtypes of hyperthyroid disease.

**Subjects and methods:**

We conducted a retrospective cohort study (September 2020-July 2023) including patients with GD (n = 577) and toxic nodular/multinodular goiter (TNG/ TMNG) (n = 216) who had available TPOAb measurements and thyroid ultrasonography. Within the GD cohort, multivariable logistic regression was used to assess associations of TPOAb titer with nodules and DTC.

**Results:**

TPOAb positivity was more frequent in GD than in TNG/TMNG (60.4% vs. 7.4%, p < 0.001). Among GD patients, thyroid nodules were detected in 25.8% and were less frequent in TPOAb-positive patients compared with TPOAb-negative patients (8.3% vs. 52.6%, p < 0.001). Within GD, DTC was rare among those without nodular disease (0.93%) but substantially higher in nodular GD (14.1%, p < 0.001), exceeding the prevalence observed in TNG/TMNG (p = 0.009). TPOAb status further stratified DTC risk in GD: DTC was present in 1.15% of TPOAb-positive compared to 9.21% of TPOAb-negative patients (p < 0.001), with 84% of GD-associated DTCs arising in the TPOAb-negative subgroup. Regression analyses showed that TPOAb titer was independently associated with lower frequencies of both DTC and nodules in GD, whereas thyrotropin receptor antibodies levels showed no independent association.

**Conclusion:**

In GD, our study showed that TPOAb positivity was strongly associated with lower prevalence of thyroid nodules and DTC, whereas TPOAb-negative patients constitute a clinically relevant, higher-risk subgroup.

## INTRODUCTION

Graves' disease (GD) is an autoimmune thyroid disease characterized by excessive thyroid hormone production and unregulated thyroid enlargement due to thyrotropin receptor antibodies (TRAb) (^1^, ^2^). It is the most common cause of hyperthyroidism, affecting approximately 1.2% of people living in iodine-sufficient areas. Toxic nodular/multinodular goiter (TNG/ TMNG), another cause of hyperthyroidism, is less prevalent than GD but becomes increasingly common with age and in iodine-deficient regions (^3^). While GD is not classically considered a nodular thyroid disorder and typically demonstrates diffuse hyperfunction on scintigraphy, thyroid nodules are frequently identified on ultrasonography (USG) in up to 33-55% of cases; reported prevalence varies depending on the inclusion of subcentimeter nodules and pseudonodular changes (^4^, ^5^). Historically, GD was thought to be protective against thyroid cancer; however, GD has now been associated with a significantly increased risk of developing DTC when thyroid nodules are also present, compared with other nodular thyroid diseases (^6^, ^7^). In nodular thyroid disease, the prevalence of DTC varies by 1-5%, whereas in nodular GD it can reach up to 15%, and in surgical series varies from 22.2% to as high as 45.8% (^7^, ^8^, ^9^).

Despite strong evidence for an increased risk of thyroid cancer in GD patients with nodular disease, the predictive factors for thyroid nodule development in GD are not well defined (^6^, ^7^, ^8^). Demographic and clinical factors such as gender, age, body mass index, thyroid-stimulating hormone, and TRAb do not differ significantly between GD patients with and without thyroid cancer (^5^). Some authors have proposed that TRAb, an autoantibody specific to GD, might promote the formation of potentially malignant nodules via angiogenesis and induction of growth factors. However, no clear association between TRAb titers and the presence of nodules or DTC development has been demonstrated (^10^, ^11^, ^12^, ^13^), and some reports have even found that lower TRAb titers confer a higher risk of thyroid cancer (^14^). Furthermore, TRAb levels are known to fluctuate and may decrease or become negative with different treatment modalities (^15^).

Regarding autoimmunity, thyroid peroxidase antibodies (TPOAb) are more prevalent than thyroglobulin antibodies (TgAb) and serve as a better indicator for autoimmune thyroid disease, reflecting thyroid inflammation and thyrocyte damage (^16^, ^17^). Some surgical series have observed higher DTC frequency in thyroidectomy specimens with TPOAb positivity (^18^, ^19^), although more recently, TPOAb has been proposed as a potential mediator of protective tumor immunity (^20^, ^21^). A study of prognostic factors in individuals previously diagnosed with differentiated thyroid cancer (DTC) found that autoantibody positivity and a history of autoimmune thyroid disease were associated with reduced risk of death, metastasis, and/or recurrence (^22^). Although TPOAb is present in about 60% of GD cases, its role in GD remains unclear (^23^).

While GD-specific immunity has been postulated to explain the link between GD and cancer development, the mechanism underlying the association between GD and nodules is unknown. This study aims to investigate whether the presence of TPOAb, reflecting secondary destructive immunity in GD, is associated with the presence of thyroid nodules and the development of DTC.

## SUBJECTS AND METHODS

### Study design and setting

A retrospective cohort study was conducted in an adult endocrinology outpatient clinic. The study protocol was approved by the local ethics committee and was conducted in accordance with the Declaration of Helsinki. Due to the retrospective design, all data were obtained from routine clinical care with no additional interventions performed.

### Study population

Consecutive adult patients with hyperthyroidism, evaluated between September 2020 and July 2023, were screened using the hospital's electronic medical record system. Hyperthyroidism was diagnosed and classified as GD or TNG/TMNG according to the 2016 American Thyroid Association Guidelines (^1^). Inclusion criteria were age >18 years, availability of at least one TPOAb measurement, and at least one documented thyroid USG examination. Exclusion criteria included age <18 years, history of external neck irradiation, absence of TPOAb or USG data, fine-needle aspiration biopsy without available cytological results, or thyroidectomy without available histopathological report. Follow-up was defined as the interval from hyperthyroidism diagnosis to thyroidectomy or the last clinic visit, whichever occurred first. Of 1116 patients screened during the study period, 577 patients with GD and 216 with TNG/TMNG were included in the final analysis. The TNG/TMNG group served as a comparator cohort, representing a biologically distinct form of hyperthyroidism. TPOAb status was primarily defined using the measurement at diagnosis; if unavailable, the first available follow-up measurement was used. TPOAb was evaluated both as a binary variable (positive vs negative) and, in exploratory analyses, as a continuous titer to examine dose-response associations.

### Thyroid USG and nodule definition

Thyroid USG was performed by experienced radiologists as part of routine clinical care. For the purposes of this study, a thyroid nodule was defined as at least one discrete, well-demarcated lesion observed on USG.

### Fine-needle aspiration biopsy, thyroidectomy, and histopathology

Fine-needle aspiration biopsy was performed according to guidelines, mostly for nodules >1 cm with suspicious sonographic features, and for selected subcentimeter nodules when clinically indicated. Cytologic findings were classified according to the Bethesda System for Reporting Thyroid Cytopathology (^24^). Indications for thyroidectomy included compressive symptoms, failure or intolerance of antithyroid drugs, severe ophthalmopathy, cytologic suspicion of malignancy, or patient preference. DTC was defined by histopathology based on the eighth edition of the American Joint Committee on Cancer Tumor-Node-Metastasis system (^25^). Tumors detected incidentally on surgical pathology were classified as incidental (IDTCs). DTC cases were classified for descriptive purposes as cytology-driven DTC (Bethesda V-VI prompting surgery) or as DTC arising from nodules without Bethesda V-VI cytology, diagnosed after thyroidectomy.

### Laboratory measurements

Thyroid function tests and thyroid autoantibodies were measured by electrochemiluminescence immunoassays (Roche Diagnostics, USA). TRAb was measured by radioimmunoassay. Reference ranges were: thyroidstimulating hormone 0.35-4.94 mU/L, free T4 0.70-1.48 ng/dl, free T31.71-3.71 pg/mL.TPOAb levels ≥ 34 lU/mL and TRAb ≥0.55 IU/L were considered positive.

### Outcomes

Primary outcomes were: (^1^) the presence of at least one thyroid nodule and (^2^) histopathologically confirmed DTC. Analyses of predictors were performed within the GD cohort.

### Statistical analysis

Normality was assessed using the Kolmogorov-Smirnov test and visual inspection. Continuous variables are reported as mean ± standard deviation for normally distributed data, or as median (interquartile range) for non-normally distributed data. Categorical variables are expressed as frequencies and percentages. Group comparisons utilized Student's t-test or Mann-Whitney U test for continuous variables and Chi-square test for categorical variables, as appropriate. TPOAb status was initially analyzed as a binary variable (positive vs negative); subsequently, TPOAb titers were analyzed as a continuous variable to explore dose-response associations. Covariates were selected a priori based on biological plausibility and prior literature (age, sex, TRAb, duration of follow-up). Pearson or Spearman correlation was used as appropriate. Associations with thyroid nodules and DTC were evaluated using multivariable binary logistic regression models. Analyses were case-wise. A two-sided p-value < 0.05 was considered statistically significant. All analyses were performed using SPSS v. 20 (IBM Corp., USA).

## RESULTS

### Study population and TPOAb distribution

A total of 793 patients with hyperthyroidism (595 females, 198 males) were included. Of these, 577 had GD (438 females, 75.9%) and 216 had TNG/ TMNG (157 females, 72.7%). Demographic and laboratory characteristics of the GD and TNG/TMNG groups by TPOAb status are summarized in Table 1. TPOAb positivity was observed in 349 (60.4%) of GD patients, compared to 16 (7.4%) of TNG/TMNG patients (p < 0.001).

**Table 1:** Baseline characteristics of the study groups according to diagnosis and thyroid peroxidase antibody status

**Variable**	**Graves' disease**		*p*	**Toxic nodular/multinodular goiter**		*p*
	**TPOAb-negative (n = 228)**	**TPOAb-positive (n = 349)**		**TPOAb-negative (n = 200)**	**TPOAb-positive (n = 16)**	
Age, years	45.7 ±13.5	40.1 ±13.0	&lt;0.001	59.0 ±12.4	61.0 ±14.8	0.510
Sex (male/female), n(%)	52/176(22.8/77.2)	87/262(24.9/75.1)	0.560	55/145(27.5/72.5)	4/12(25/75)	0.830
Body mass index, kg/m^2^	26.4 ±3.6	25.5 ±3.4	0.500	26.6 ±3.8	24.8 ±4.2	0.670
Ophthalmopathy, n (%)	33(14.5)	76(21.8)	0.028	—	—	—
TSH, mlU/L	0.020(0.01-0.1)	0.010(0.01-0.07)	0.035	0.030(0.01-0.1)	0.020(0.005-0.03)	0.870
Free T4, ng/dL	1.6(1.2-2.1)	1.6(1.2-2.4)	0.100	1.4(1.2-1.94)	1.2(1.1-1.9)	0.200
Free T3, pg/mL	4.0(3.1-5.5)	4.3(3.2-7.2)	0.520	3.7 (3.0-4.9)	4.4(2.9-7.3)	0.390
TRAb, IU/L	8.4 ±8.9	15.2 ± 13.4	&lt;0.001	**—**	**—**	**—**

TPOAb: thyroid peroxidase antibody; TRAb: thyrotropin receptor antibody; TSH: thyroid-stimulating hormone. Values are presented as mean ± standard deviation or median (interquartile range), as appropriate. *p*-Values indicate comparisons according to TPOAb status within each diagnostic group.

### TPOAb association with thyroid nodules in GD

Among GD patients, at least one thyroid nodule on USG was detected in 149 (25.8%). Thyroid nodules were significantly less frequent in TPOAb-positive compared with TPOAb-negative GD patients (8.3% [29/349] vs 52.6% [120/228], p < 0.001). Frequency by TPOAb status is shown in **Figure 1.**

**Figure 1:**
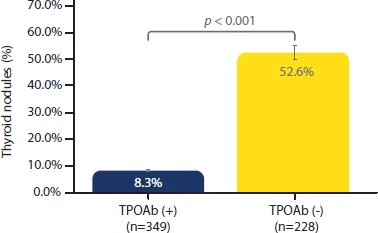
Frequency of thyroid nodules according to thyroid peroxidase antibody (TPOAb) status in Graves' disease (GD). Among patients with GD, thyroid nodules on ultrasonography were significantly less frequent in TPOAb-positive patients than TPOAb-negative patients.

### DTC in hyperthyroidism and GD

DTC was detected in 37 (4.67%) of all patients with hyperthyroidism, including 12 (5.55%) of 216 TNG/ TMNG patients and 25 (4.33%) of 577 GD patients (*p* > 0.05). There was no significant difference in overall DTC prevalence between GD and TNG/ TMNG. Nevertheless, within GD, both nodular and TPOAb status significantly affected DTC prevalence **(Figure 2)**. Among GD patients, DTC was uncommon in patients without thyroid nodules (0.93% [4/428]), albeit significantly more frequent in those with nodules (14.1% [21/149]; p < 0.001). Additionally, there was a significantly higher rate of DTC in patients with nodular GD than the TNG/TMNG group (14.1% vs. 5.55% [12/216]; *p* = 0.009), as summarized in **Table 2.**

**Figure 2:**
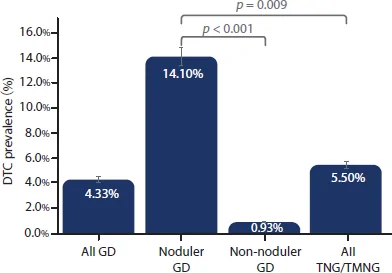
Prevalence of differentiated thyroid cancer (DTC) according to disease type and nodular status. The prevalence of DTC was low in Graves' disease (GD) without thyroid nodules, markedly increased in nodular GD, and intermediate in toxic nodular or multinodular goiter (TNG/TMNG).

**Table 2:** Thyroid nodules and differentiated thyroid cancer according to disease type

**Outcome**	**Graves' disease (n = 577)**	**TNG/ TMNG (n = 216)**	*P*
Thyroid nodules, n (%)	149(25.8)	216(100)	***—***
Differentiated thyroid cancer, n (%)			
Total	25(4.33)	12(5.55)	0.560
Without nodules	4/428 (0.93)	**—**	**—**
With nodules	21/149(14.1)	5.6(12/216)	0.009

TNG/TMNG: toxic nodular/multinodular goiter. Percentages are calculated within each disease group.

### Impact of TPOAb status on DTC in GD

TPOAb status further stratified DTC prevalence within the GD cohort. DTC was detected in 4 (1.15%) of 349 GD/TPOAb-positive patients, whereas 21 (9.21%) of 228 GD/TPOAb-negative patients had DTC (p < 0.001). In the GD population, 84% of all DTC cases occurred in the TPOAb-negative group. The distribution of DTC according to TPOAb status among patients with GD is shown in **Figure 3**.

**Figure 3:**
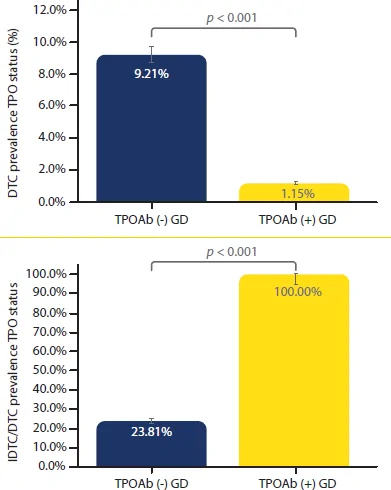
Distribution of differentiated thyroid cancer (DTC) according to thyroid peroxidase antibody (TPOAb) status in patients with Graves' disease (GD). The lower panel shows the proportion of incidental DTC (IDTC)/DTC.

### Surgical enrichment and thyroidectomy findings

Thyroidectomy was performed in 56 patients with GD (9.7%); among these patients, 25 (44.6%) were diagnosed with DTC. Of the 228 GD/TPOAb-negative patients, 26 (10.96%) underwent thyroidectomy, and DTC was detected in 21 patients (80.8%). Among these 21 patients, 16 underwent surgery due to suspicious or malignant cytology (Bethesda V-VI), whereas 5 were operated on for other indications. In the GD population, patients with nodular GD who were TPOAb-negative had the highest risk of developing DTC, at 17.5% (21/120) **(Figure 4**). In contrast, among the 349 GD/TPOAb-positive patients, 30 (8.59%) underwent thyroidectomy, with DTC detected in 4 patients (13.3%) **(Figure 4**). Indications for surgery in this subgroup included severe Graves' orbitopathy in two patients and a large goiter in two patients. All DTC cases in the TPOAb-positive subgroup were classified as IDTC. Among the 216 patients with TNG/TMNG, 53 (24.5%) underwent thyroidectomy, and DTC was diagnosed in 12 patients (22.6%) **(Figure 4**). Of these, 5 patients underwent surgery for compressive symptoms, and IDTC was identified upon postoperative histopathological examination.

**Figure 4:**
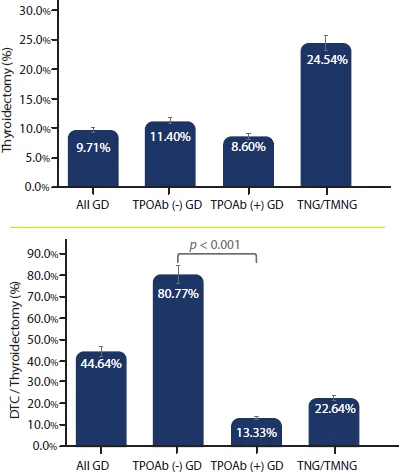
Frequency of thyroidectomy and proportion of differentiated thyroid cancer (DTC) among operated patients according to thyroid peroxidase antibody (TPOAb) status in Graves' disease (GD), compared with toxic nodular or multinodular goiter (TNG/TMNG). The upper panel shows the proportion of patients who underwent thyroidectomy within each diagnostic group. The lower panel shows the proportion of differentiated thyroid cancer among patients who underwent thyroidectomy in each group.

### Correlation and regression analyses in GD

Correlation analyses in patients with GD demonstrated that both TPOAb positivity and TPOAb titers were negatively correlated with DTC, whereas neither TRAb titers nor TRAb positivity correlated with DTC **(Table 3).** In multivariable binary logistic regression analyses limited to GD, higher TPOAb titers were independently associated with lower odds of DTC. When predictors of thyroid nodules were analyzed, age emerged as an independently positive association, whereas TPOAb titer was an independent negative predictor. TRAb levels and follow-up duration were not independently associated with either thyroid nodules or **DTC (Table 4).**

**Table 3:** Parameters correlated with nodules and differentiated thyroid cancer in Graves' disease

**Variable**	**Differentiated thyroid cancer**		**Nodule**	
	**r**	*P*	***r***	***p***
Sex	0.040	0.310	0.550	0.190
Age	-0.030	0.380	0.220*	**&lt;0.001**
Graves' disease	-0.010	0.790	-0.410	0.330
TRAb^#^	-0.030	0.470	-0.180*	**&lt;0.001**
TP0Ab^#^	-0.130*	**&lt;0.001**	-0.470*	**&lt;0.001**
TRAb^#^(1/0)	-0.020	0.720	-0.510	0.250
TPOAb^#^(1/0)	-0.150*	**&lt;0.001**	-0.500*	**&lt;0.001**
Follow-up period	0.020	0.560	-0.010	0.820
Nodule in Graves'disease	0.250*	**&lt;0.001**	—	—

TRAb: thyroid receptor antibody; TPOAb: thyroid peroxidase antibody; Bold values indicate statistical significance; *significance at *p &lt;* 0.001 ^#^antibody level at the time of diagnosis.

**Table 4:** Binary logistic regression analysis to investigate the factors affecting differentiated thyroid cancer and nodules in Graves' disease

**Dependent variable**	**Independent variable**	***β***	*P*	**OR**	**OR (95% Cl)**	
					**Lower**	**Upper**
DTC in GD	Sex	0.310	0.640	1.360	0.380	4.890
	Age	-0.036	0.089	0.970	0.930	1.010
	TP0Ab^#^	-0.030	0.040*	0.997	0.995	1.000
	TRAb^#^	-0.07	0.770	0.990	0.950	1.040
	Follow-up period	0.003	0.570	1.003	0.990	1.010
Nodules in GD	Sex	0.290	0.340	1.340	0.730	2.430
	Age	0.028	0.004*	1.029	1.009	1.048
	TP0Ab^#^	-0.005	&lt;o.oo1*	0.995	0.993	0.996
	TRAb^#^	-0.017	0.200	0.980	0.960	1.010
	Follow-up period	0.001	0.850	1.001	0.995	1.010

CI: confidence interval; DTC: differentiated thyroid cancer; GD: Graves' disease; OR: odds ratio TPOAb: thyroid peroxidase antibody; TRAb: thyrotropin receptor antibody. TPOAb titers were analyzed as a continuous variable: *statistically significant at *p&lt;* 0.05. ^#^antibody level at the time of diagnosis.

## DISCUSSION

In this large group of GD patients, both those who underwent surgery and those who did not, TPOAb positivity was strongly and inversely associated with the presence of thyroid nodules and DTC. This indicates that both conditions were less prevalent in TPOAb-positive individuals. In contrast, no association was observed between TRAb titers and either nodule formation or DTC. The finding that 93% of patients in the TNG/TMNG group were TPOAb-negative supports the association between lack of autoimmune activity and nodular thyroid disease. In GD, the presence of thyroid nodules significantly increased the prevalence of DTC (reaching 14% in our study). The high rate of cancer in nodular GD observed in our cohort aligns with previous reports, which have found cancer in approximately 15% of individuals with nodular GD (^8^, ^9^). This rate is also higher than both the rate observed in our control TNG/TMNG cohort and that reported in the general population with nodular thyroid disease (^7^). Additionally, DTC is more common in nodular GD than in TNG/TMNG supports the hypothesis that the microenvironment in GD may facilitate thyroid cancer development. In our cohort, disease duration, sex, age, and TRAb were not independent predictors of DTC. Consistent with our findings, Keskin and cols. (^14^) identified the presence of nodules as an independent predictor of thyroid cancer in surgically treated GD.

Although Hashimoto's thyroiditis (HT) has often been implicated in DTC pathogenesis, available evidence remains conflicting (^18^, ^19^, ^26^, ^27^). Some surgical series have identified TgAb as a positive marker for DTC, whereas others reported increased cancer risk only when both TPOAb and TgAb coexist in the setting of elevated thyroid-stimulating hormone levels (^19^, ^20^). Importantly, TPOAb seropositivity has not consistently emerged as an independent predictor of DTC across studies (^19^, ^26^-29). In general, cytological studies have not shown an increased risk of thyroid cancer in patients with HT compared to surgical series, and some reports even describe a decreased risk in individuals with HT (^30^, ^31^). Recent surgical data indicate that tumors grow more quickly in DTC patients who are TPOAb-negative, supporting the hypothesis that thyroid autoimmunity may have a protective effect against cancer (^32^). In a multicenter, multinational study, Paparodis and cols. (^20^) showed an inverse linear relationship between TPOAb levels and DTC risk. TPOAb and TgAb are recognized as markers of destructive and cytotoxic autoimmunity involved in HT pathogenesis (^27^). While TPOAb has been recognized for its cytotoxic effect on DTC, it remains unproven that TgAb shows a similar effect (^18^). In HT patients, steadily increasing TPOAb levels cause thyrocyte death and gland shrinkage. This process may inhibit the growth of both benign and malignant thyroid lesions over time due to autoimmune cytotoxicity (^20^, ^27^). In the literature, DTC in patients with chronic lymphocytic thyroiditis is characterized by a distinct immune infiltrate, rich in macrophages, CD3+, CD8+, CD20+, and FoxP3+ lymphocytes (^33^, ^34^, ^35^, ^36^). Differentiating between lymphocytes encircling a tumor and those attributable to thyroiditis-induced infiltration is challenging. In some cases, focal immune infiltrates are mistakenly seen as direct evidence of a link between autoimmunity and cancer development instead of a secondary immune response to tumor presence (^37^). Concomitant thyroiditis is associated with smaller tumor size, reduced angioinvasion, and a lower incidence of lymph node metastasis in DTC, suggesting a less aggressive disease phenotype (^22^, ^38^, ^39^).

Both GD and HT are autoimmune thyroid diseases, although they arise from fundamentally different pathogenic mechanisms. In GD, proliferative signaling is predominant, whereas cytotoxic immune activity dominates in HT (^40^). In our cohort, TPOAb positivity occurred in approximately 60% of GD patients, consistent with previous studies (^16^, ^17^, ^23^).

The prevalence of DTC was significantly lower in GD/TPOAb-positive patients compared to GD/ TPOAb-negative patients (1.2% vs. 9.21%). The specific role of TPOAb in GD remains unclear, albeit high TPOAb activity may slow the progression to overt nodular disease by limiting the clinical manifestation of latent or early malignant foci in GD. Imam and cols. (^26^, ^28^) found a higher risk of DTC in both GD and HT when TPOAb titers were low or absent, supporting our interpretation and representing a key finding of this study. Conversely, the presence of TRAb has been associated with thyroid cancer risk in GD (^10^, ^11^, ^12^, ^13^, ^14^). In our cohort, GD/TPOAb-positive patients exhibited higher TRAb levels than GD/TPOAb-negative patients, although a lower prevalence of DTC. Previous studies did not stratify patients according to TPOAb status (^10^, ^11^, ^12^, ^13^, ^14^). Additionally, no association between TRAb and DTC was found in the study by Ergin and cols. (^13^). Our findings suggest that risk stratification for DTC in GD is more accurately achieved by considering TPOAb status than TRAb levels. Both TRAb-mediated proliferative signaling and TPOAb-mediated autoimmune destructiveness may change the structure and biology of thyroid tissue in GD, which often shows diffuse heterogeneity, pseudonodular changes, and incidentally detected IDTC that may remain clinically silent unless surgery is performed (^27^, ^28^, ^41^). In contrast, the absence of TPOAb may allow relatively unopposed nodular development, contributing to the higher observed DTC prevalence in this subgroup.

In the entire GD cohort, thyroidectomy was performed in 9.7% of patients, with a slightly higher surgical rate in GD/TPOAb-negative patients (10.96%) than GD/TPOAb-positive patients (8.59%). In published surgical series of patients with GD, the reported prevalence of DTC varies by 22.2-45.8% (^8^, ^9^, ^10^). In our cohort, DTC was detected in 25 of the 56 patients (44.6%) who underwent thyroidectomy for GD, consistent with the literature. Importantly, thyroidectomy was not performed at random but rather based on clinical indications, most commonly cytological suspicion, large compressive goiter, or severe orbitopathy. Consequently, the thyroidectomy subgroup constitutes a clinically enriched, higher-risk population, and cancer prevalence in operated patients should not be generalized to the entire GD population. Accordingly, our main conclusions are drawn from group-level analyses stratified by TPOAb status and the presence of thyroid nodules. As expected, the presence of thyroid nodules was the principal driver of surgical referral in GD. This accounts for the high proportion of cancerous specimens among surgically treated patients and corroborates previous findings that nodules are the principal risk factor for DTC in GD (^12^). Although GD/TPOAb-positive patients were younger than GD/ TPOAb-negative patients, DTC prevalence remained higher in GD/TPOAb-negative patients than in the older TNG/TMNG group. While age was an independent factor associated with nodular disease, TPOAb status was more relevant for DTC prevalence.

Paparodis and cols. (^21^) reported that IDTCs are more frequently detected in patients with HT than in those with GD. In our cohort, all DTC cases observed in the GD/TPOAb-positive group were IDTCs, identified on postoperative histopathological examination following thyroidectomy performed for non-malignant indications. Similarly, in the multicenter study by Premoli and cols. (^42^), DTCs ≥ 1 cm in GD were associated with less favorable prognostic features, whereas the presence or absence of GD did not impact the prognosis of detected IDTC. In summary, our findings indicated that the risk of DTC decreases with TPOAb positivity, and that detected DTCs in these patients are predominately IDTCs with low recurrence rates and excellent long-term prognosis, thus representing cases of limited clinical significance (^21^, ^42^, ^43^).

This study has several limitations. Patients without available TPOAb measurements or thyroid USG data were excluded, potentially introducing selection bias. Thyroidectomy was performed according to real-world, indication-based clinical practice; thus, surgical patients represent a special group with higher pre-test probability of malignancy. The cancer rate observed in the thyroidectomy group should therefore not be extrapolated to the entire GD population. Data on iodine status, smoking status, and family history of thyroid cancer were not available for the GD or control groups; however, all patients were drawn from the same geographic region. Lastly, long-term oncologic follow-up was unavailable; hence, the associations between GD, TPOAb status, and DTC could not be evaluated prospectively.

A major strength of this study is the inclusion of a large, consecutive real-world cohort of patients with GD followed in an endocrinology outpatient setting, as opposed to a surgically selected cohort. To our knowledge, this is the first study to demonstrate a statistically significant negative association between TPOAb positivity and both thyroid nodules and DTC in GD, while directly comparing these findings with TNG/TMNG patients, a biologically distinct form of hyperthyroidism.

In conclusion, previous research has established that nodular GD is associated with a higher risk of DTC than other nodular thyroid disorders. Our findings expand on this by demonstrating that the prevalence of both thyroid nodules and DTC is the highest in GD patients who are TPOAb-negative. In GD, TPOAb positivity has been independently associated with a lower frequency of thyroid nodules and DTC. The molecular mechanisms underlying this association remain to be elucidated. Based on our findings, TPOAb status may serve as a clinically significant marker for risk stratification of nodules and nodule-derived DTC in GD, potentially guiding more personalized management strategies for patients with GD.

## Data Availability

datasets related to this article will be available upon request to the corresponding author.

## References

[1] Ross DS, Burch HB, Cooper DS, Greenlee MC, Laurberg P, Maia AL (2016). 2016 American Thyroid Association Guidelines for Diagnosis and
Management of Hyperthyroidism and Other Causes of
Thyrotoxicosis. Thyroid.

[2] Bartalena L, Chiovato L, Vitti P (2016). Management of hyperthyroidism due to Graves’ disease: frequently
asked questions and answers (if any). J Endocrinol Invest.

[3] Abraham-Nordling M, Byström K, Törring O, Lantz M, Berg G, Calissendorff J (2011). Incidence of hyperthyroidism in Sweden. Eur J Endocrinol.

[4] Cantalamessa L, Baldini M, Orsatti A, Meroni L, Amodei V, Castagnone D (1999). Thyroid nodules in Graves disease and the risk of thyroid
carcinoma. Arch Intern Med.

[5] Soares MN, Borges-Canha M, Neves C, Neves JS, Carvalho D (2023). The role of Graves’ disease in the development of thyroid nodules
and thyroid cancer. Eur Thyroid J.

[6] Papanastasiou A, Sapalidis K, Goulis DG, Michalopoulos N, Mareti E, Mantalovas S (2019). Thyroid nodules as a risk factor for thyroid cancer in patients
with Graves’ disease: A systematic review and meta-analysis of observational
studies in surgically treated patients. Clin Endocrinol (Oxf).

[7] Durante C, Hegedüs L, Czarniecka A, Paschke R, Russ G, Schmitt F (2023). 2023 European Thyroid Association Clinical Practice Guidelines
for thyroid nodule management. Eur Thyroid J.

[8] Kraimps JL, Bouin-Pineau MH, Mathonnet M, De Calan L, Ronceray J, Visset J (2000). Multicentre study of thyroid nodules in patients with Graves’
disease. Br J Surg.

[9] Belfiore A, Russo D, Vigneri R, Filetti S (2001). Graves’ disease, thyroid nodules and thyroid
cancer. Clin Endocrinol (Oxf).

[10] Song Y, Fu L, Wang P, Sun N, Qiu X, Li J (2020). Effect of Graves’ disease on the prognosis of differentiated
thyroid carcinoma: a meta-analysis. Endocrine.

[11] Tam AA, Kaya C, Kiliç FB, Ersoy R, Çakir B (2014). Thyroid nodules and thyroid cancer in Graves’
disease. Arq Bras Endocrinol Metabol.

[12] Palella M, Giustolisi FM, Modica Fiascaro A, Fichera M, Palmieri A, Cannarella R (2023). Risk and Prognosis of Thyroid Cancer in Patients with Graves’
Disease: An Umbrella Review. Cancers (Basel).

[13] Ergin AB, Saralaya S, Olansky L (2014). Incidental papillary thyroid carcinoma: clinical characteristics
and prognostic factors among patients with Graves’ disease and euthyroid
goiter, Cleveland Clinic experience. Am J Otolaryngol.

[14] Keskin C, Sahin M, Hasanov R, Aydogan BI, Demir O, Emral R (2019). Frequency of thyroid nodules and thyroid cancer in
thyroidectomized patients with Graves’ disease. Arch Med Sci.

[15] Laurberg P, Wallin G, Tallstedt L, Abraham-Nordling M, Lundell G, Tørring O (2008). TSH-receptor autoimmunity in Graves’ disease after therapy with
anti-thyroid drugs, surgery, or radioiodine: a 5-year prospective randomized
study. Eur J Endocrinol.

[16] Carvalho GA, Perez CL, Ward LS (2013). The clinical use of thyroid function tests. Arq Bras Endocrinol Metabol.

[17] Balucan FS, Morshed SA, Davies TF (2013). Thyroid autoantibodies in pregnancy: their role, regulation and
clinical relevance. J Thyroid Res.

[18] Xiao Y, Zhou Q, Xu Y, Yuan SL, Liu QA (2019). Positive thyroid antibodies and risk of thyroid cancer: A
systematic review and meta-analysis. Mol Clin Oncol.

[19] Wu X, Lun Y, Jiang H, Gang Q, Xin S, Duan Z (2014). Coexistence of thyroglobulin antibodies and thyroid peroxidase
antibodies correlates with elevated thyroid-stimulating hormone level and
advanced tumor stage of papillary thyroid cancer. Endocrine.

[20] Paparodis R, Livadas S, Karvounis E, Bantouna D, Zoupas I, Angelopoulos N (2023). Elevated Preoperative TPO Ab Titers Decrease Risk for DTC in a
Linear Fashion: A Retrospective Analysis of 1635 Cases. J Clin Endocrinol Metab.

[21] Paparodis RD, Karvounis E, Bantouna D, Chourpiliadis C, Chourpiliadi H, Livadas S (2020). Incidentally Discovered Papillary Thyroid Microcarcinomas Are
More Frequently Found in Patients with Chronic Lymphocytic Thyroiditis Than
with Multinodular Goiter or Graves’ Disease. Thyroid.

[22] Souza SL, Montalli Da Assumpção LV, Ward LS (2003). Impact of previous thyroid autoimmune diseases on prognosis of
patients with well- differentiated thyroid cancer. Thyroid.

[23] DeGroot LJ, De Groot LJ, Chrousos G, Dungan K, Feingold KR, Grossman A, Hershman JM (2000). Endotext.

[24] Cibas ES, Ali SZ (2017). The 2017 Bethesda System for Reporting Thyroid
Cytopathology. Thyroid.

[25] Tuttle RM, Haugen B, Perrier ND (2017). Updated American Joint Committee on Cancer/Tumor-Node-Metastasis
Staging System for Differentiated and Anaplastic Thyroid Cancer (Eighth
Edition): What Changed and Why?. Thyroid.

[26] Imam S, Paparodis R, Sharma D, Jaume JC (2014). Lymphocytic profiling in thyroid cancer provides clues for
failure of tumor immunity. Endocr Relat Cancer.

[27] Azizi G, Keller JM, Lewis M, Piper K, Puett D, Rivenbark KM (2014). Association of Hashimoto’s thyroiditis with thyroid
cancer. Endocr Relat Cancer.

[28] Imam S, Paparodis RD, Rafiqi SI, Ali S, Niaz A, Kanzy A (2022). Thyroid Cancer Screening Using Tumor-Associated DN T Cells as
Immunogenomic Markers. Front Oncol.

[29] Qin J, Yu Z, Guan H, Shi L, Liu Y, Zhao N (2015). High Thyroglobulin Antibody Levels Increase the Risk of
Differentiated Thyroid Carcinoma. Dis Markers.

[30] Anil C, Goksel S, Gursoy A (2010). Hashimoto’s thyroiditis is not associated with increased risk of
thyroid cancer in patients with thyroid nodules: a single-center prospective
study. Thyroid.

[31] Jankovic B, Le KT, Hershman JM (2013). Clinical Review: Hashimoto’s thyroiditis and papillary thyroid
carcinoma: is there a correlation?. J Clin Endocrinol Metab.

[32] Veit F, Graf D, Momberger S, Helmich-Kapp B, Ruschenburg I, Peters A (2017). Papillary Thyroid Cancer and Coexisting Autoimmune
Thyroiditis. Horm Metab Res.

[33] Holm LE, Blomgren H, Löwhagen T (1985). Cancer risks in patients with chronic lymphocytic
thyroiditis. N Engl J Med.

[34] Cunha LL, Morari EC, Guihen AC, Razolli D, Gerhard R, Nonogaki S (2012). Infiltration of a mixture of immune cells may be related to good
prognosis in patients with differentiated thyroid carcinoma. Clin Endocrinol (Oxf).

[35] Sulaieva O, Selezniov O, Shapochka D, Belemets N, Nechay O, Chereshneva Y (2020). Hashimoto’s thyroiditis attenuates progression of papillary
thyroid carcinoma: deciphering immunological links. Heliyon.

[36] Maimela NR, Liu S, Zhang Y (2018). Fates of CD8+ T cells in Tumor Microenvironment. Comput Struct Biotechnol J.

[37] Boi F, Pani F, Mariotti S (2017). Thyroid Autoimmunity and Thyroid Cancer: Review Focused on
Cytological Studies. Eur Thyroid J.

[38] Dos Santos Valsecchi VA, Betoni FR, Ward LS, Cunha LL (2024). Clinical and molecular impact of concurrent thyroid autoimmune
disease and thyroid cancer: From the bench to bedside. Rev Endocr Metab Disord.

[39] Marotta V, Sciammarella C, Chiofalo MG, Gambardella C, Bellevicine C, Grasso M (2017). Hashimoto’s thyroiditis predicts outcome in intrathyroidal
papillary thyroid cancer. Endocr Relat Cancer.

[40] Fröhlich E, Wahl R, Fahey T (2015). Updates in the Understanding and Management of Thyroid Cancer.

[41] Alhubaish ES, Alibrahim NT, Mansour AA (2023). The Clinical Implications of Anti-thyroid Peroxidase Antibodies
in Graves’ Disease in Basrah. Cureus.

[42] Premoli P, Tanda ML, Piantanida E, Veronesi G, Gallo D, Masiello E (2020). Features and outcome of differentiated thyroid carcinoma
associated with Graves’ disease: results of a large, retrospective,
multicenter study. J Endocrinol Invest.

[43] Lee J, Nam KH, Chung WY, Soh EY, Park CS (2008). Clinicopathologic features and treatment outcomes in
differentiated thyroid carcinoma patients with concurrent Graves’
disease. J Korean Med Sci.

